# Cost-effectiveness analysis of colonoscopy and fecal immunochemical testing for colorectal cancer screening in China

**DOI:** 10.3389/fpubh.2022.952378

**Published:** 2022-08-12

**Authors:** Yinan Ren, Mingye Zhao, Dachuang Zhou, Qian Xing, Fangfang Gong, Wenxi Tang

**Affiliations:** ^1^School of International Pharmaceutical Business, China Pharmaceutical University, Nanjing, China; ^2^Center for Pharmacoeconomics and Outcomes Research of China Pharmaceutical University, Nanjing, China; ^3^Department of Hospital Group Office, Shenzhen Luohu Hospital Group Luohu People's Hospital (The Third Affiliated Hospital of Shenzhen University), Shenzhen, China

**Keywords:** colorectal cancer, screening, electronic colonoscopy, fecal immunochemical testing, economic evaluation

## Abstract

**Objective:**

This study aimed to evaluate the cost-effectiveness of the colorectal cancer screening in China, and that when the screening was implemented in a specific region.

**Methods:**

A 13-state Markov model was established to compare four screening protocols, including annual fecal immunochemical testing (FIT1), biennial fecal immunochemical testing (FIT2), electronic colonoscopy every 10 years (e-CSPY10), and electronic colonoscopy every 5 years (e-CSPY5), with no screening from the perspective of Chinese healthcare system. The model simulated the health states of a cohort of 100,000 average-risk individuals aging from 50 to 75. Additionally, scenarios including the implementation in a specific region, starting from 40, and incompletely successful treatment of cancer were also analyzed.

**Results:**

Annual and biennial FIT could save 8.13USD (US Dollar) and 44.96USD per person, and increase 0.0705QALYs (Quality-Adjusted Life Years) and 0.2341 QALYs compared with no screening, respectively. Annual FIT could decrease costs by 36.81USD per person and increase 0.1637 QALYs in comparison to biennial FIT. The results showed that both annual and biennial FIT for screening were dominant over no screening, and annual FIT was dominant over biennial FIT. The ICER (Incremental Cost-Effectiveness Ratio) for e-CSPY10 were 1183.51USD/QALY and 536.66USD/QALY compared with FIT1 and FIT2. The ICER for e-CSPY5 were 1158.16USD/QALY and 770.85USD/QALY compared with FIT1 and FIT2. And the ICER for e-CSPY5 relative to e-CSPY10 was 358.71USD/QALY. All the ICER values were lower than the economic threshold of 2021 Chinese GDP (Gross Domestic Product) per capita in 2021(12554.42USD).

**Conclusions:**

It is worthwhile to popularize CRC screening in mainland China, as FIT always saving costs and colonoscopy is cost-effective. Regions with high income can take electronic colonoscopy every 10 years, or even every 5 years into consideration when determining the specific strategies.

## Introduction

Colorectal cancer (CRC) is currently one of the most common and fatal cancers worldwide. In 2018, there were approximately 1.93 million new cases globally, ranking third in cancer incidence. Simultaneously, the number of deaths caused by CRC worldwide was approximately 940,000, ranking second among all cancers (1). In China, CRC was listed fourth and fifth in incidence and mortality in 2015, with 89,993 new cases and 44,361 deaths. The world standard rates of incidence and mortality were ~17.12/100,000 and ~7.85/100,000 (2), respectively. It is estimated that there will be 642,300 cases of CRC in China and 221,100 deaths by 2025 (3). The incidence of CRC increases substantially after the age of 50, peaking around 75 to 80 years old. CRC not only seriously deceases the quality of life but also poses a considerable economic burden. The average costs for the diagnosis and treatment of CRC in China have increased by 6.9 to 9.2% per year, and the personal medical expenses of patients within 1 year of a new diagnosis accounted for approximately 60% of their household income (4).

CRC is associated with various factors, including inflammatory bowel disease, family history of CRC, age over 50 years old, male gender, obesity, type 2 diabetes mellitus, unhealthy living habits, and gut microbiota (5–9). Most CRCs originate from precancerous lesions, which are mainly referred to as polyps. This process begins with abnormal crypts that evolve into polyps and eventually develop into cancer, taking about 10–15 years (10). In addition, the larger the diameter of the polyps, the more villi, and the higher the degree of atypicality, the higher the risk of canceration (11, 12). Moreover, polyps cause hematochezia, gastralgia, and abdominal distension, reducing the quality of life of patients (13). Endoscopic surgeries are usually performed to resect cancerous polyps, whereas adequate surveillance is used to prevent cancer. A systematic analysis showed that the incidence and mortality of CRC after screening were reduced by 40 and 60%, respectively, indicating that early screening combined with timely diagnosis and treatment is an effective method to reduce the disease burden (14).

The screening and diagnostic methods commonly used in China mainly include colonoscopy, fecal immunochemical testing (FIT), sigmoidoscopy, colon computed tomography imaging, and multi-target fecal FIT-DNA detection, with colonoscopy being the gold standard. Moreover, colonoscopy including optical colonoscopy and electronic colonoscopy, with the latter one providing detailed contrast enhancement of the mucosal surface and blood vessel. Additionally, there are no significant differences between the effectiveness of these two technologies in CRC screening. But the unit cost of optical colonoscopy is lower than electronic colonoscopy. In light of this, this study would take electronic colonoscopy as an example to assess the cost-effectiveness of colonoscopy (15, 16). Besides, FIT is commonly used in CRC screening and diagnosis as it is non-invasive and low-cost. There are a lot of researches on the effectiveness of sigmoidoscopy for CRC screening in European and American countries. However, the application of sigmoidoscopy for cancer screening is not common in China, and the guidelines of China not recommending this technology for mass screening in most regions. Additionally, although colon computed tomography imaging is non-invasive and highly sensitive, it is limited for mass screening for its strict requirements for bowel preparation, the lacking of inspection equipment and specialists, and the risk of radiation. Also, multi-target fecal FIT-DNA detection has not been widely used in mass screening because its effectiveness is still need to be confirmed in China, and it is an expensive method which requires central laboratories (17).

The economic evaluations of various strategies in developed countries, such as the United States, the United Kingdom, and France, have demonstrated the cost-effectiveness of screening. However, the results of these studies may not be suitable for developing countries, and relative evaluations are lacking in mainland China. We used index terms containing “colon cancer,” “colorectal cancer,” “cost effectiveness,” “cost utility,” “cost benefit,”“economic evaluation,”“CEA,” “CUA,” “CBA,” “China,” and “Chinese” and searched PubMed, Embase, CNKI, and other databases, identifying fewer than 17 studies on screening (18–34). Moreover, the outcome indicators used in these studies were distinct, and the only few economic evaluations did not use incremental cost-effectiveness analysis, resulting in incomparability with international studies.

This study aimed to assess the usefulness and cost-effectiveness of CRC screening in China and analyze the impact of regions, screening frequency, starting age, and therapeutic effect of cancer treatment on the results. We selected Luohu District, Shenzhen, one of the pre-eminent cities in China as a sample to explore the suitable strategies at the district level. Luohu Hospital Group began implementing the Institution-based Colorectal Cancer Screening Program (I-CRCSP) in 2018, and continued the project annually. The office-working group over 40 years old and retired people younger than 75 years old are screened using electronic colonoscopy (e-CSPY), with females taking account of 52.65% and the average age of the participants being about 53, which was in the range of the starting age recommended by several guidelines. This project is also a pioneer in CRC screening in China, and we predict that the evidence of its effectiveness and cost-effectiveness is of great importance. Moreover, this study would compare the e-CSPY with FIT as it was most common used in CRC screening.

## Materials and methods

### Study design

Considering that the guidelines of China and USPSTF (United States Preventive Services Taskforce) both recommend the average-risk population who is over 50 to participate CRC screening until 75 (17, 35, 36), this study set the target subjects to enter the model from 50 and exit when he/she is 75.

### Screening strategies

The guideline of USPSTF recommends the screening strategies involving all the subsistent methods and corresponding frequencies. However, considering the real situation of China, the screening methods evaluated in this study were FIT and e-CSPY. In addition, it is recommended by the Chinese Journal of Oncology for general population need to be performed every 5 to 10 years and FIT test need to be used each year (37). Therefore, this study analyzed the cost-effectiveness of the colonoscopy at the upper and lower limits of the recommended time range, with e-CSPY was repeated every 10 years(e-CSPY10) and 5 years(e-CSPY5), and FIT being repeated annually (FIT1) and biennially (FIT2).

FIT are immunoassays specific for human hemoglobin, forming an antibody-antigen complex with its globin moiety (38). Usually, one or two stool samples are collected for tests without diet restriction. Those whose results of FIT are positive need to undergo a colonoscopy for diagnosis. FIT has replaced gFOBT (guaiac-based fecal occult blood test) as the Fecal detection technology nowadays. However, the sensitivity of FIT for the detection of precancer is limited, even that for the discovery of cancer is high.

E-CSPY is widely used for full colorectal examinations and treatment. The electron camera probe at the front of the colonoscopy transmits images of the colon mucosa to the processing center, and the pictures are displayed on the monitor screen. Intestinal preparation is required a few h before the examination. Patients are asked to drink laxative until water is excreted. The examination requires general anesthesia. First, the patient is positioned in a left-sided or prone knee-flex position. The colonoscope is passed through the rectum, descending colon, spleen flex, transverse colon, hepatic curvature, and ascending colon in sequence and finally reaches the cecum (39). The physician quickly advances the colonoscope into the intestine and observes the intestinal epithelium while slowly withdrawing. The induction takes approximately 4 min, and the withdrawal takes at least 6 min.

Endoscopic resection should be performed whenever the morphological structure of polyps permits (40). Pedunculated polyps are generally removed (41). For sessile or flattened polyps at risk of pT1 cancer, which represents the earliest form of clinically relevant cancer and is the key stage of tumor sequence, surgery is required to completely remove the lesion (42). Specifically, it is necessary to select the appropriate excision methods according to the diameter of the polyp (17, 43–45). Moreover, intraoperative or postoperative bleeding due to resection may require routine hemostasis or additional endoscopic management (46).

In terms of the screening project in Shenzhen, e-CSPY was encouraged before the annual physical examination of the office-working group and carried out with the exam simultaneously. Cancerous patients were excluded based on the Hospital Information System and face-to-face interviews in advance, and the screening was conducted in tertiary hospitals belongs to Luohu Hospital Group. Since 2020, 5,343 participants have been screened, and the misdiagnosed rate was approximately 19.39% (47). The misdiagnosed rate was the number of missed polyps divided by the total number of polyps found in biannual examination. This figure was derived from the retrospective data collected in 250 patients from July, 2007 to July, 2012 of the gastrology department in Shenzhen Luohu hospital. For the purpose of exploring the impact of implement site which reflecting the distinction of factors such as baseline characteristics of population and economy level, this study also simulated scenario where subjects in Shenzhen were tested using FIT, rather than undergoing e-CSPY only.

### Model overview

#### Natural history and Markov model

CRC may develop from three paths: (1) the adenoma-carcinoma sequence, in which the disease progresses according to the sequence of normal epithelial cells, low-risk adenomas, high-risk adenomas, and cancers (48); (2) serrated lesions, mainly referring to hypertrophic polyps and sessile serrated adenomas, both characterized by the serrated structure of the upper part of the crypt, but only the sessile serrated adenoma is cancerous; and (3) de novo, indicating that the cancer starts from the normal colon mucosa (49). Among them, CRC developing from the de novo pathway accounts for <5%, whereas the proportion of cancer originating from serrated lesions is unknown, ranging from 5 to 30%. Additionally, including these two paths would complicate the model. Therefore, we considered the adenoma-carcinoma sequence only (50).

In this study, low-risk adenoma is defined as polyps with a diameter < 10 mm, and high-risk adenoma refers to polyps with a diameter >10 mm or containing more than 25% of the villous structure. The TNM classification of cancer grades was applied.

A Markov model was designed to simulate the disease history of a cohort of 100,000 subjects. The model was proposed and validated by Wong et al. in 2015. We adjusted the model structure and assumptions to match the population of mainland China (see Key assumptions section for details). The health states contained normal, low-risk adenoma, high-risk adenoma, CRC I, CRC II, CRC III, CRC IV, and death. In addition, false positive state was set to reflect the misdiagnosis of the screening technologies. Of note, carcinomatosis was divided into preclinical cancer and post-diagnosis cancer. Preclinical cancer refers to a state in which a patient is asymptomatic but has cancer. And adenomas were removed once detected, and patients returned to normal. High-risk adenomas have a certain probability of progressing to cancer each year.

The model cycle was 1 year, and subjects in each cycle progressed according to the path or were stable when proceeding to the next cycle. Moreover, subjects in all states may die. The CRC-caused mortality was only applied to individuals with cancer, whereas natural mortality was applied to the entire target population.

**Figure 1:**
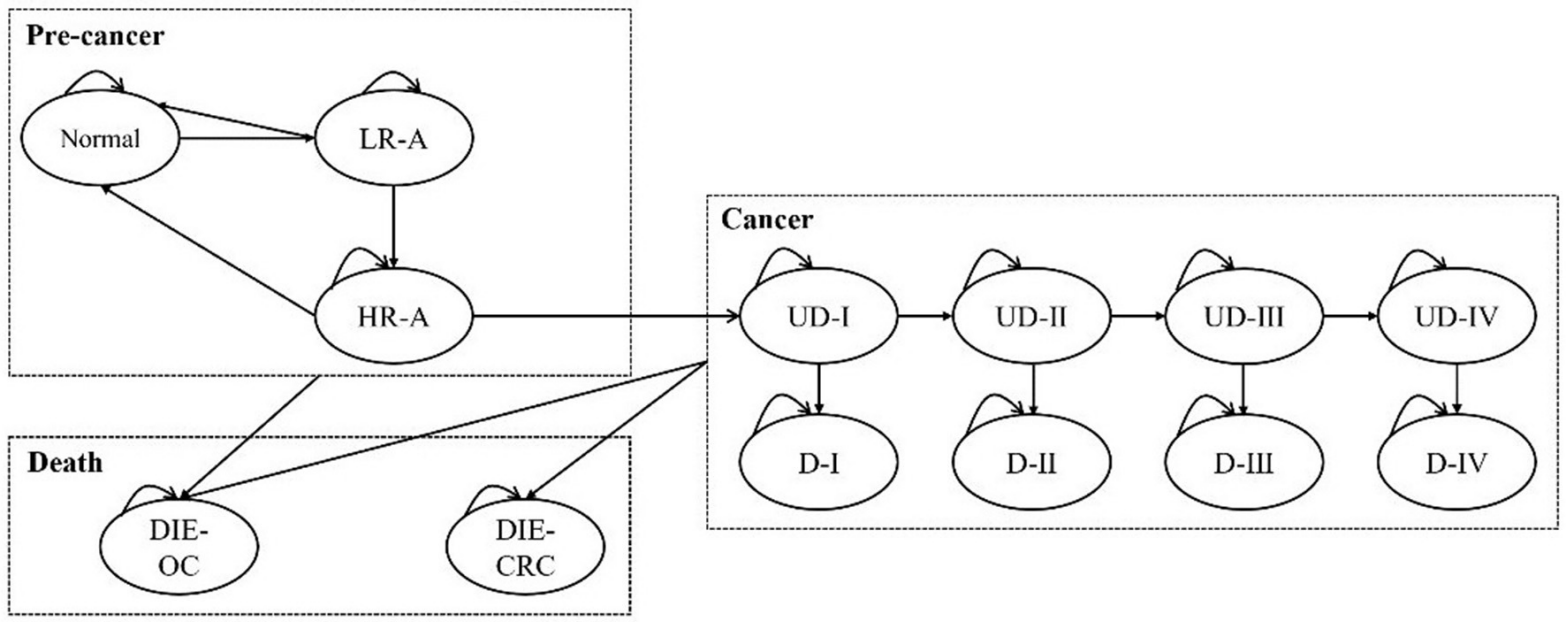
The Markov model for the base case analysis. L/HR-A, low/high-risk adenoma; (U)D-I/II/III/IV, (undiagnosed) cancer at stage I/II/III/IV; DIE-CRC, die of colorectal cancer; DIE-OC, die due to other causes.

CRC patients may be diagnosed through clinic visits when they notice symptoms or through screening. Those who participate in screening but are not diagnosed may also be diagnosed through the first route. The natural history of subjects in the screening scenario is the same as those in the scenario without screening. The main difference is that early screening may prevent disease progression at the adenoma stage, and early treatment might also suppress cancer development. Consequently, the number of subjects in each state is different between the two scenarios, ultimately resulting in the distinction of the cost and health benefits. The model was implemented in Excel, and the schematic of the model is shown in Figure 1.

#### Key assumptions

1. Assuming that the initial state of screening and non-screening scenarios is the same, and the original number of subjects were calculated according to the distribution of disease. Additionally, the number of diagnosed cancer patients is initially zero.
2. Considering that the risk of progression is closely related to the diameter, degree of villous components, and atypicality of adenomas, it is assumed that the disease progresses step by step. For example, low-risk adenoma first progresses to high-risk adenoma and then progresses to cancer. Similarly, high-risk adenoma only progresses to stage I cancer (the first adjustment of the model).
3. We assumed that preclinical cancer patients receive specific treatments according to cancer stages and stop progressing as soon as they are diagnosed.
4. Although screening using colonoscopy may lead to fatal adverse events, such as perforation and bleeding, the probability is extremely small according to expert opinions and literature [perforation 0.01% (51); bleeding 0.22% (17)]. Therefore, death due to adverse events was not considered in this study (the second adjustment of the model).
5. Patients are expected to undergo colonoscopy examinations when they visit doctors. Moreover, this study assumed that the visit rate of adenoma was 0 and that of CRC IV was 100% on the basis of expert interviews.
6. Subjects in false positive state were assumed to receive treatment only for 1 year, and would be back to the normal state in the next cycle.
7. It was assumed that those whose results of FIT were positive but hadn't undergo colonoscopy would not be treated unless they were symptomatic.

### Model parameters

#### Epidemiological data

The number of initial states was calculated based on the prevalence of adenomas and CRC. The prevalence of adenomas was calculated based on the proportion of low-/high-risk adenomas, excluding hypertrophic polyps in Hong Kong and adenomas in mainland China (13, 52). The prevalence of CRC was based on a study on CRC disease burden in China, which used 2017 global burden of disease data to estimate the incidence, prevalence, and mortality of CRC in China from 1990 to 2017 (53). The proportion of patients in CRC stages I to IV was based on data from Hong Kong in 2019 and estimated after excluding patients who could not be graded (54). The prevalence of adenoma and CRC in Luohu District, Shenzhen was calculated by combining the local detection rate and the data mentioned above.

#### Transition probability

The transition probability from normal to cancer stemmed from a systematic review of natural history models of CRC in China (55). The transition probabilities between various stages of cancer were used only for preclinical states because the development of cancer stops upon diagnosis. This is mainly because although cancers may progress during treatment in the real world, the probabilities will vary over time, as observed from the Kaplan–Meier curves in randomized controlled trials. However, the memoryless property of the Markov model means that it is not possible to distinguish the duration of different individuals being diagnosed in the same state, resulting in the inapplicability of dynamic transition probabilities.

#### Mortality

Mortality included age-specific natural mortality and cancer-specific mortality. The natural mortality rate was according to the China population and Employment Statistics Yearbook in 2017 (56). The cancer-specific mortality was based on a study in Hong Kong, which conducted an economic evaluation of CRC Screening in Asia.

#### Utility

The health-related quality of life of patients originated from a study in Hong Kong, which used SF-6D (Short Form health state classification and utility scoring system based on 6 dimensions) to measure the utility score of 151 adenoma patients and 364 CRC patients (57). At the same time, we set the score to 1 for healthy individuals and 0 for those who died.

#### Screening-related parameters

This section includes participation rate of screening, sensitivity and specificity of FIT and e-CSPY, and excision rate of polyps. The screening participation rate was calculated based on the program in Shenzhen and the performance parameters of screening technologies derived from published literature, with the hypothesis that the specificity of e-CSPY is 100% (50). Furthermore, we assumed that the excision rate was also 100% after interviews with experts. See Supplementary Table A; Table 1 for details.

**Table 1:** Age-specific natural mortality (56).

**Age**	**Mortality**
40–44	0.18%
45–49	0.26%
50–54	0.42%
55–59	0.62%
60–64	1.03%
65–69	1.72%
70–74	3.06%

#### Costs

The health system perspective was applied when evaluating costs; therefore, only the direct medical costs were incorporated in the model. Screening costs included program fees, examination fees of FIT and e-CSPY, polypectomy fees, histopathology examination fees, and follow-up costs. This study hypothesized that all polyps would be removed and examined by pathology, followed by surveillance within 1 year. The costs of adenoma treatment included medical service expenses, diagnosis and examination fees, surgery fees, and surveillance costs. The cost of CRC treatment derived from a study on the economic burden of CRC in Chinese patients in 2017 and was the average expense during the first year after diagnosis (58). The cost of cancer treatment in Shenzhen was based on a study of disease burden in Guangzhou (31), a first-tier city in Guangdong Province. Furthermore, CRC patients are required to receive follow-up surveillance after treatment, according to the “Chinese Protocol of Diagnosis and Treatment of Colorectal Cancer (59),” including routine medical examinations and imaging examinations, and the costs referred to the prices in Shenzhen. It was assumed that the follow-up surveillance stopped 5 years later (59). All prices were converted to 2021 prices at a discount rate of 5% and shown in USD (US Dollar), with 1USD=6.45CNY (Chinese Yuan). See Table 2 for details.

**Table 2:** Cost parameters.

	**Parameters**	**Value(USD)**	**Lower value(USD)**	**Upper value(USD)**	**Distribution**	**Resource**
Treatment	Low-risk adenoma	593.32	444.99	741.65	Gamma	Calculation
	High-risk adenoma	593.32	444.99	741.65	Gamma	Calculation
	CRC I^a^	11,000.47	8,250.35	13,750.59	Gamma	(58)
	CRC II^a^	12,237.85	9,178.38	15,297.31	Gamma	
	CRC III^a^	13,041.75	9,781.31	16,302.19	Gamma	
	CRC IV^a^	14,225.68	10,669.26	17,782.09	Gamma	
	CRC-follow up	1,907.80	1,430.85	2,384.74	Gamma	Calculation
Treatment in Shenzhen	CRC I^a^	13,273.92	9,955.44	16,592.40	Gamma	(31) (60)
	CRC II^a^	17,796.91	13,347.68	22,246.13	Gamma	
	CRC III^a^	21,667.11	16,250.33	27,083.88	Gamma	
	CRC IV^a^	33,530.59	25,147.94	41,913.23	Gamma	
Screening	Project (total cost)	141,085.27	105,813.95	176,356.59	Gamma	Calculation
	FIT	2.64	1.98	3.29	Gamma	File
	Electronic colonoscope/person	140.72	105.54	175.90	Gamma	File
	Excision/person	283.36	212.52	354.19	Gamma	File
	Pathological examination/person	28.53	21.40	35.66	Gamma	File
	Follow up/person	140.72	105.54	175.90	Gamma	File

^a^CRC I/ II/ III/ IV, colorectal cancer at stage I/ II/ III/ IV.

### Sensitivity analysis

The sensitivity analysis was conducted to verify the uncertainty of parameters. We explored the impact of each parameter through deterministic sensitivity analysis and presented the results in the form of a tornado figure. We also carried out a probability sensitivity analysis with net monetary benefits as intermediate indicators. A Monte Carlo simulation was conducted to draw from the distributions of parameters randomly for 10,000 iterations. The probability and utility values followed the Beta distribution, and costs followed the gamma distribution. Finally, the results were presented as scatter plots and cost-effective acceptability curves.

### Scenario analysis

We considered three scenarios to verify the model uncertainty.

**Figure 2:**
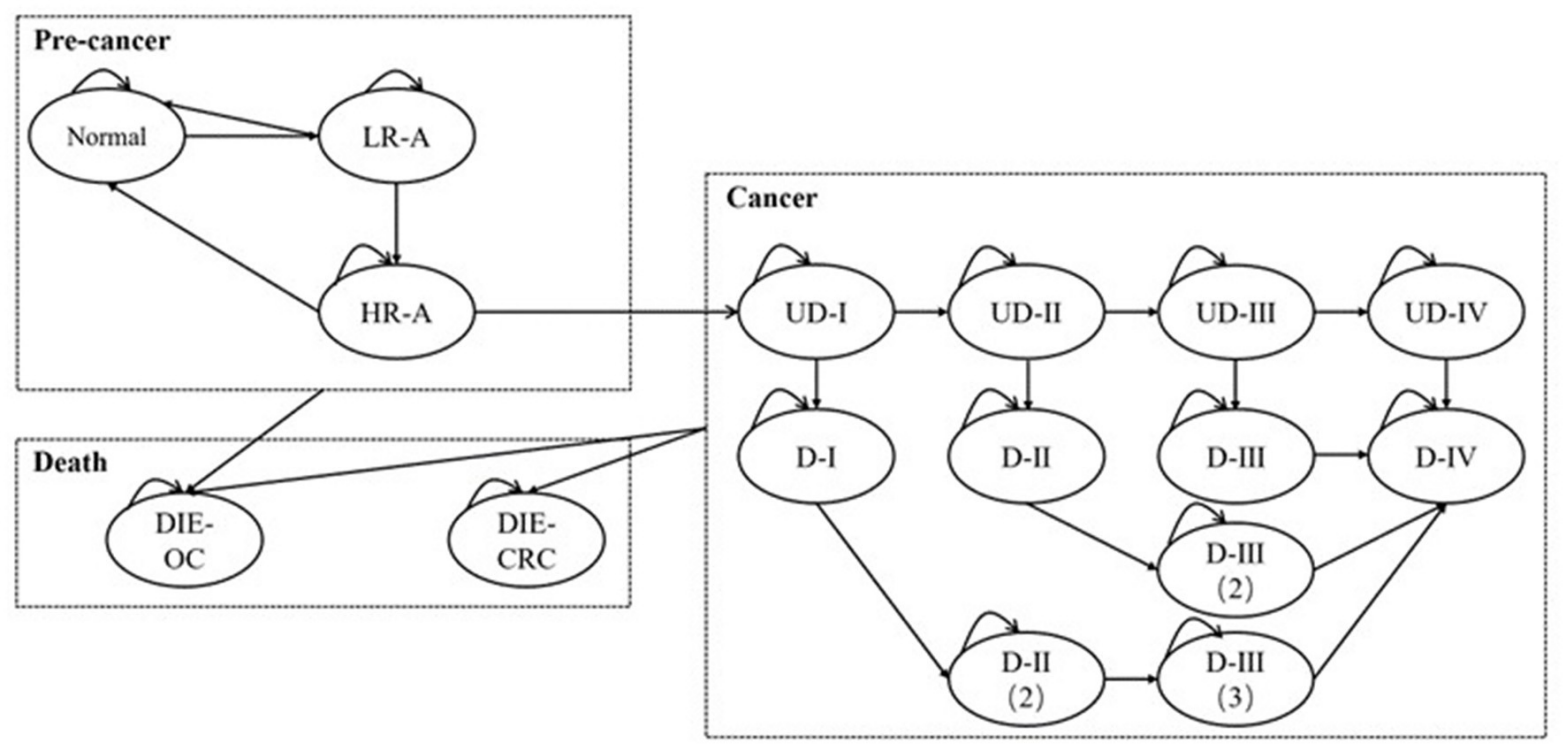
The Markov model for analysis in scenario 3. L/HR-A, low/high-risk adenoma; (U)D-I/II/III/IV, (undiagnosed) cancer at stage I/II/III/IV; DIE-CRC: die of colorectal cancer; DIE-OC, die due to other causes.

Scenario 1 Evaluate the cost-effectiveness of CRC screening when the setting is Shenzhen and compare the results with those in China to explore potential factors.
Scenario 2 Set the age of the participants entering the model to 40 and simulate to 75 years old or death in Chinese population to analyze the impact of the starting age on the cost-effectiveness of the screening program.
Scenario 3 Assuming medical treatments cannot completely inhibit the progression of cancers. In real world, cancerous patients may continue to worsen when receiving therapeutic treatment, but the probability of metastasis may lower than that in preclinical stages. However, because of the inapplicability of the time-varying transition probabilities in Markov model, we used stable values for analysis. Additionally, it was assumed that the more times patients receive treatments, the greater the transition probabilities are. The parameters are presented in detail in Table 3, and the structure of the model in scenario 3 is shown in Figure 2.

**Table 3:** Parameters in scenario 3.

	**Parameters**	**Value**	**Lower value**	**Upper value**	**Distribution**	**Resource**
Transition probability	CRC I-II^a^	25%	18.75%	31.25%	Beta	Assumption
	CRC II-III^a^	35%	26.25%	43.75%	Beta	Assumption
	CRC II-III (from I)^b^	40%	30.00%	50.00%	Beta	Assumption
	CRC III-IV^a^	35%	26.25%	43.75%	Beta	Assumption
	CRC III-IV (from II)^b^	40%	30.00%	50.00%	Beta	Assumption
	CRC III-IV (from I)^b^	45%	33.75%	56.25%	Beta	Assumption

^a^CRC I/ II/ III/ IV, colorectal cancer at stage I/ II/ III/ IV. ^b^CRC II-III (from I), individuals who transit from stage II cancer to stage III cancer who were diagnosed with stage I cancer initially (the rest states in the similar manner are explained by analogy).

Additionally, this study evaluated the effectiveness of screening in avoiding advanced cancers and deaths, and the indicators were CRC cases and deaths being prevented. The CRC cases and deaths were the aggregated value of patients suffering from cancer at stage four and those died at the endpoint of the simulation.

## Results

### Base case analysis

The results of base case analysis showed that both annual and biennial FIT were dominant over non-screening scenario, as well as the comparison with annual FIT and biennial FIT. More precisely, FIT1 and FIT2 saved 8.13USD and 44.96USD per person, and increased 0.0705QALYs (Quality-Adjusted Life Years) and 0.2341QALYs in comparison with no screening. And FIT1 could save 36.81USD per person and increase 0.1637QALYs vs. FIT2.

Strategies of e-CSPY10 and e-CSPY5 increased costs by 39.14USD and 141.84USD per person, and increased 0.3052QALYs and 0.3954QALYs, with the ICER (Incremental Cost-Effectiveness Ratio) being 128.24USD/QALY and 358.73USD/QALY compared with no screening.

The ICER for e-CSPY10 were 1183.51USD/QALY and 536.66USD/QALY compared with FIT1 and FIT2, respectively. And that for e-CSPY5 were 1158.16USD/QALY and 770.85USD/QALY, respectively. In addition, the ICER for e-CSPY5 relative to e-CSPY10 was 358.71USD/QALY.

All of the ICERs were lower than economic threshold of DGP (Gross Domestic Product) per capita of China in 2021(12554.42USD).

See Table 4 for details.

**Table 4:** Results of the base case analysis.

	**FIT1**	**FIT2**	**e-CSPY10**	**e-CSPY5**
Incremental costs (USD)				
FIT1^*^	–	–	84.10	178.67
FIT2^*^	−8.13	–	75.96	178.67
e-CSPY10^*^	–	–	–	102.71
e-CSPY5^*^	–	–	–	–
No screening	−44.96	−36.81	39.14	141.84
Incremental QALYs				
FIT1^*^	–	–	0.0,711	0.1,613
FIT2^*^	0.0,705	–	0.1,415	0.2,318
e–CSPY10^*^	–	–	–	0.0,902
e-CSPY5^*^	–	–	–	–
No screening	0.2,341	0.1,637	0.3,052	0.3,954
ICER (CNY/QALY)				
FIT1^*^	–	–	1,183.51	1,158.16
FIT2^*^	−115.41(dominant)	–	536.66	770.85
e-CSPY10^*^	–	–	–	1,138.19
e-CSPY5^*^	–	–	–	–
No screening	−192.01(dominant)	−225.00(dominant)	128.24	358.71

^*^ FIT1, Annual FIT; FIT2, Biennial FIT; e-CSPY10, colonoscopy every 10 years; e-CSPY5, colonoscopy every 5 years.

### Sensitivity analysis

**Figure 3:**
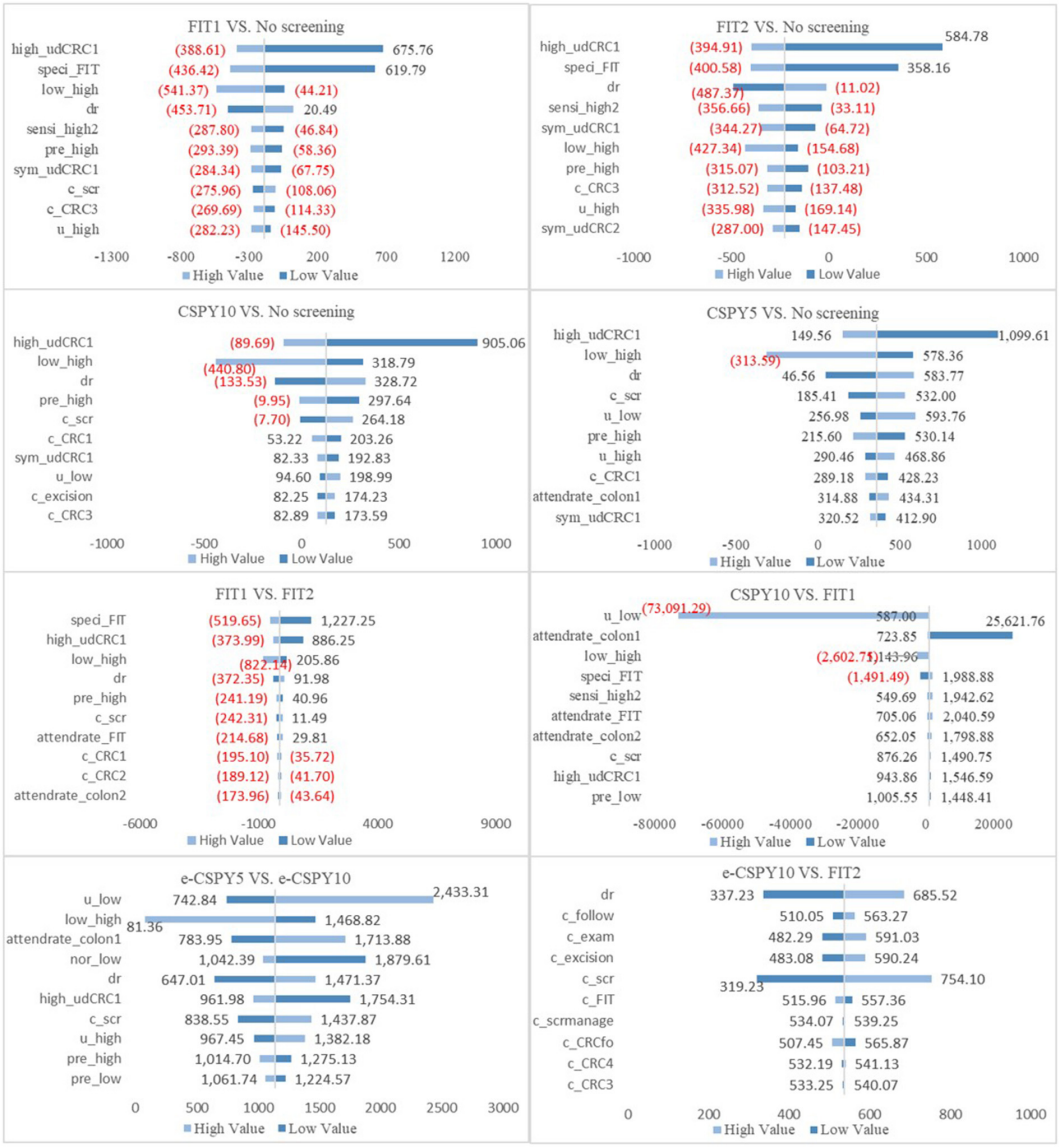
The tornado figures of Deterministic Sensitivity Analysis. High, high-risk adenoma; low, low-risk adenoma; nor, normal; udCRC, undiagnosed colorectal cancer; CRC12/3/4, colorectal cancer I/II/III/IV; speci, specificity; sensi, sensitivity; dr, discount rate; c, cost; scr, screening; pre, prevalence rate; colon1, colonoscopy; colon2, colonoscopy following FIT; scrmanage, the management of the screening; E-CSPY10/5, electronic colonoscopy every 10/5 years; FIT1/2, annual/ biennial FIT.

Deterministic sensitivity analysis showed that the ICER was lower than the threshold value when the parameters varied separately, indicating that the results of base case analysis were robust. When the control group is no screening, the transition probability from high-risk adenoma to undiagnosed CRC was the most sensitive parameter. When compare e-CSPY10 with annual FIT, the sensitivity and specificity of the technologies and attendance rates of screening may influence the results. Additionally, the cost-effectiveness may be impacted by the disease progress, utility of patients, participation rates, and the fees of colonoscopy when comparing e-CSPY at different intervals. See Figure 3 for details.

**Figure 4:**
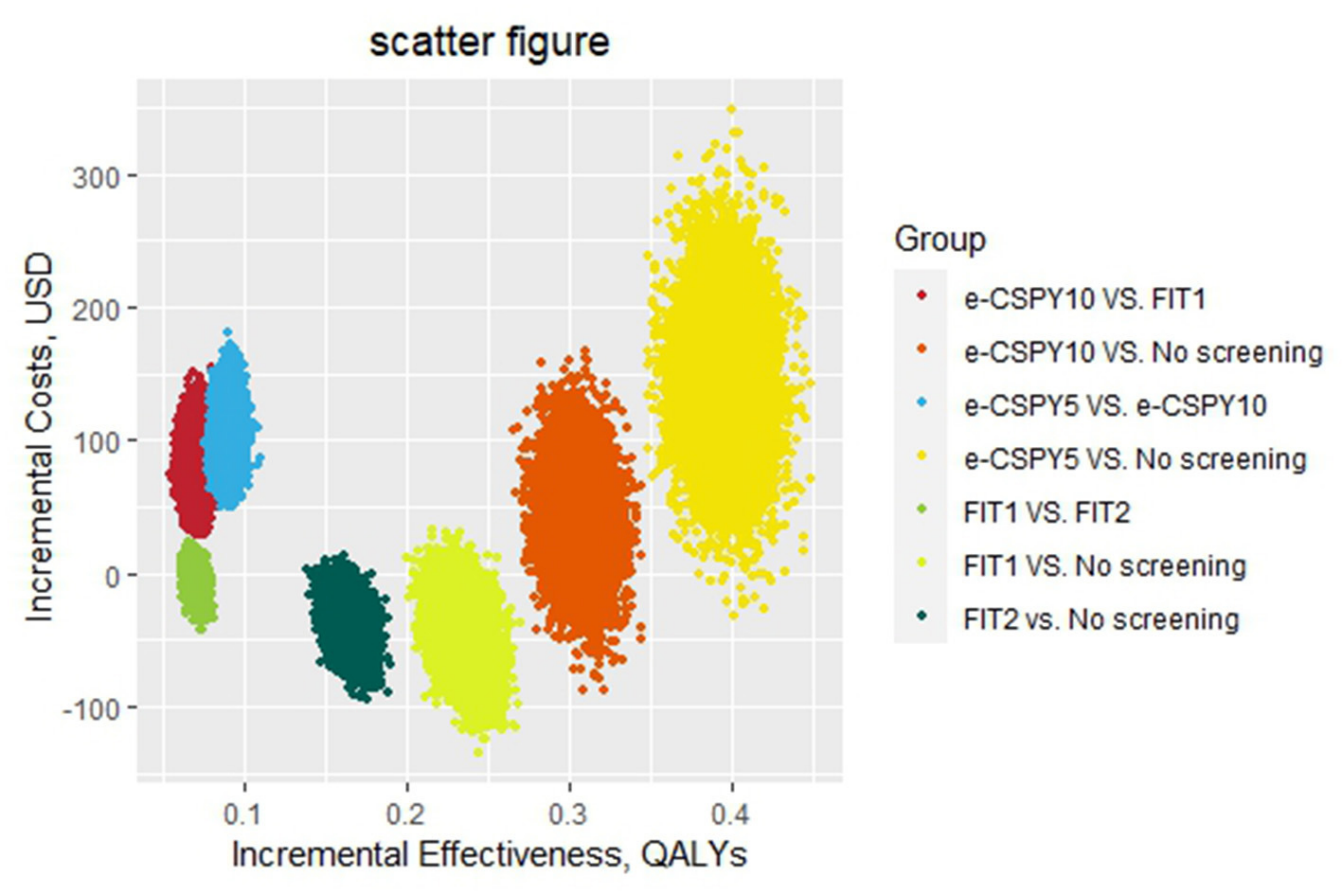
The scatter figure of the probabilistic sensitivity analysis. E-CSPY10/5, electronic colonoscopy every 10/5 years; FIT1/2, annual/ biennial FIT.

**Figure 5:**
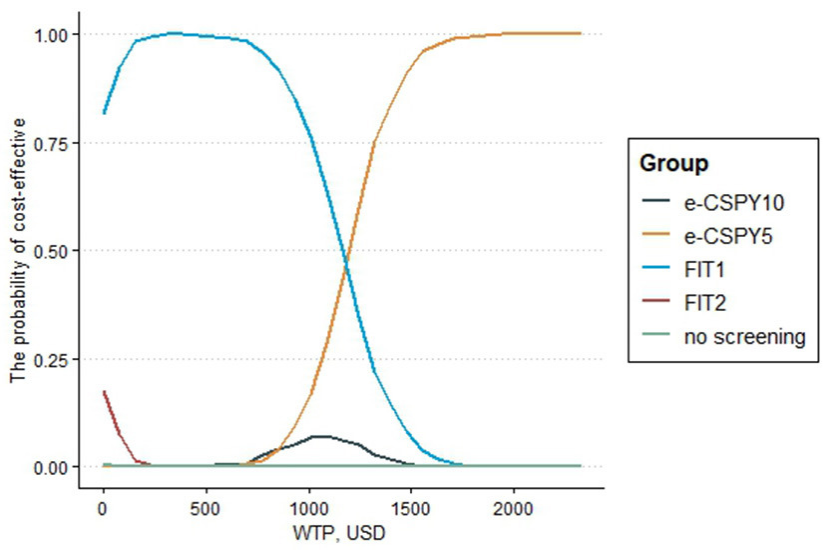
The cost-effectiveness acceptability curve. E-CSPY10/5, electronic colonoscopy every 10/5 years; FIT1/2, annual/ biennial FIT; WTP, willingness to pay.

The results of the probabilistic sensitivity analysis showed that the probability that the screening was cost-effective of FIT1 and e-CSPY5 was the same at the WTP of 1240.31USD. Annual FIT was most likely to be cost-effective when the WTP was less than 1240.31USD, and E-CSPY every 5 years was the optimal choice when the threshold was over 1240.31USD. FIT1 was the most cost-effective strategy mainly due to its low costs, and e-CSPY5 became the most cost-effective tactics may due to its excellent effectiveness in screening. Additionally, the cost-effectiveness of FIT2 wasinferior to FIT1 may on account of the slightly inferior effectiveness for CRC screening, even though it was inexpensive. Likewise, the effect of e-CSPY10 was not up to e-CSPY5 resulting in its disadvantages when the WTP was high despite of the relatively lower costs. See Figures 4, 5 for details.

### Scenario analysis

Table 5 showed the results of scenario analysis.

**Table 5:** Results of the cost-effectiveness analysis in different scenarios.

	**FIT1**	**FIT2**	**e-CSPY10**	**e-CSPY5**
ICER (USD/QALY) in Scenario 1	
FIT1	–	–	1,203.57	958.45
FIT2	−665.71(dominant)	–	273.71	459.82
e-CSPY10	–	–	–	759.86
e–CSPY5	–	–	–	–
No screening	−783.25(dominant)	−833.42(dominant)	−322.14(dominant)	−81.22(dominant)
ICER (USD/QALY) in Scenario 2	
FIT1	–	–	1325.01	1113.34
FIT2	−182.19(dominant)	–	495.85	691.76
e-CSPY10	–	–	–	975.45
e-CSPY5	–	–	–	–
No screening	−319.64(dominant)	−374.48(dominant)	−8.64(dominant)	212.76
ICER (USD/QALY) in Scenario 3	
FIT1	–	–	−345.37(dominant)	132.52
FIT2	−1082.37(dominant)	–	−691.84(dominant)	−235.05(dominant)
e-CSPY10	–	–	–	589.80
e-CSPY5	–	–	–	–
No screening	−766.25(dominant)	−617.08(dominant)	−654.53(dominant)	−384.3(dominant)

Scenario 1 When the setting of screening was Shenzhen, e-CSPY10 and e-CSPY5 became dominant over no screening. Also, ICERs for e-CSPY10 in comparison to FIT and ICER for e-CSPY5 relative to FIT and e-CSPY were lower than that in base case analysis.
Scenario 2 When the starting age of screening was brought forward to 40, e-CSPY10 became dominant over no screening. ICERs for e-CSPY10 and e-CSPY5 were lower than that in base case analysis, except the value in the comparison between e-CSPY10 and annual FIT.
Scenario 3 Considering that treatment does not completely inhibit the progression of cancer, all the strategies were dominant over no screening. In addition, e-CSPY10 was cost-saving compared with FIT. E-CSPY5 was dominant over biennial FIT. And ICER for e-CSPY5 were much lower than economic threshold compared with annual FIT and e-CSPY10.

Additionally, screening could reduce CRC cases by 1589–4497 and deaths by 1036–2537 in the long run. The number of CRC cases and deaths being avoided mentioned above were the range of the values used in other screening scenarios. Besides, the more frequent the screening and the earlier the starting age, the greater the number of cancers and deaths could be prevented. See Table 6 for details.

**Table 6:** Number of colorectal cancer patients and deaths in all scenarios.

	**Number of CRC patients**					**Number of deaths**				
	**FIT1**	**FIT2**	**e-CSPY10**	**e-CSPY5**	**No screening**	**FIT1**	**FIT2**	**e-CSPY10**	**e-CSPY5**	**No screening**
China 50–75	1,681 (↓2,918)	2,537 (↓2,062)	1,543 (↓3,056)	918 (↓3,681)	4,599	29,478 (↓1,259)	29,701 (↓1,036)	29,763 (↓974)	29,555 (↓1,182)	30,737
Shenzhen 50–75	1,798 (↓3183)	2,725 (↓2256)	1,636 (↓3,345)	970 (↓4,011)	4,981	29,456 (↓1380)	29,700 (↓1136)	29,768 (↓1068)	29,540 (↓1296)	30,836
China 40–75	2,112 (↓4,313)	3,315 (↓3,110)	1,928 (↓4,497)	1,044 (↓5,381)	6,425	3,1072 (↓2,222)	3,1432 (↓1,862)	3,1507 (↓1,787)	3,1161 (↓2,133s)	3,3294
Incompletely successful treatment	539 (↓2,069)	1,019 (↓1,589)	669 (↓1,939)	282 (↓2,326)	2,608	30,621 (↓2,107)	31,219 (↓1,509)	30,637 (↓2,091)	30,191 (↓2,537)	32,728

The symbol “↓” representing the nuber of cancer cases or deaths which may be avoided by these screening strategies.

## Discussion

Colorectal cancer has posed a great threat to human life, and screening has been proved to be a effective solution to decrease the disease burden. Countries around the world developed guidelines of screening for colorectal cancer one by another. For instance, both the guidelines of USPSTF and China listed the existing technologies for screening and diagnosis such as colonoscopy, fecal immunochemical testing (FIT), sigmoidoscopy, colon computed tomography imaging, and multi-target fecal FIT-DNA detection. This study evaluated the effectiveness and cost-effectiveness of the commonly used methods for mass screening in China, namely FIT and electronic colonoscopy in the hope of providing a reference for public health decision-making.

This study found that screening for colorectal cancer in China was cost-effective and conducive to reduce cancer cases and deaths, no matter the method was FIT or electronic colonoscopy. Moreover, annual FIT and biennial FIT were always cost saving in comparison with no screening, regardless of the starting age, screening frequency or therapeutic effect of cancer. And when the setting of screening was Shenzhen, or the inhibition of cancer treatment was incomplete, both e-CSPY10 and e-CSPY5 became dominant over no screening. Besides, e-CSPY10 could save costs and yield more QALY gains when the starting age was 40. Furthermore, optical colonoscopy would be more cost-effective compared with e-CSPY based on the results, due to the similar effectiveness of screening and lower cost.

In addition, e-CSPY was a cost-effective technology for CRC screening, no matter of the screening interval, compared with FIT. The QALY gained from screening with e-CSPY was more than that from screening using FIT, while the implementation of e-CSPY cost more, with the ICER was lower than the economic threshold.

We also found that the results were sensitive to factors such as transition probabilities, characteristics of screening technologies and costs of screening and cancer treatment, indicating the role of the screening in terms of preventing cancers and saving relevant expenses. Specifically, adenomas are usually asymptomatic and neglected, but they are at high risk of progressing to CRC within 5 to 10 years. The higher the transition probabilities and prevalence of diseases, the more CRCs may be prevented by screening. In addition, in case of the relatively low cost of screening and polypectomy, as well as high cost of cancer treatment, the increased expenditure incurred by the screening program would be low while the cost saving of cancer treatment yielding from screening would be high. Furthermore, the more accurate the instruments are, the less expenses would be waste, contributing to more benefits brought from screening. Thus, conducting screening program would be more cost-effective. As expected, a higher compliance of screening would be in favor to the cost-effectiveness of e-CSPY when compared with FIT. This may due to that more cancer cases and death could be prevented, further saving the subsequent high costs of treatment in this circumstance. This suggests the significance of improving the compliance of screening with colonoscopy considering it is invasive and time-consuming.

Moreover, a high frequency of screening and early age of first screening increased health benefits. The ICER increased as the screening frequency improved. In contrast, early screening could decrease the ICER. However, the ICER was less than the threshold value in all cases. In addition, cancer therapy outcomes were demonstrated to affect the cost-effectiveness of screening. Ideally, specific treatments should completely inhibit the progression of cancer, but 30 to 50% of patients relapse after surgeries or systemic chemotherapy (61–64) In this case, screening is more advantageous. This can be explained by the fact that screening prevents more advanced cancers and deaths at this time, thereby saving costs and increasing health benefits. Therefore, the economic evaluations based on the complete cure hypothesis may underestimate the cost-effectiveness of screening (30, 32).

There were numerous studies which proved the cost-effectiveness of FIT and colonoscopy. The results of this study are consistent with the previous evaluations. For instance, M. Aronsson et al. found that in Sweden, both FIT and colonoscopy were cost-effective strategies compared with no screening, and repeated and single screening with colonoscopy were more cost-effective than FIT in the long run (65). Nelya Melnitchouk et al. proved that screening with FIT or colonoscopy could save money and improve health compared with no screening and colonoscopy every 10 years was a superior choice in Ukraine (66). Wong et al. demonstrated in 2015 that screening with annual FIT was the optimal strategy among annual/biennial g-FOBT, annual/biennial FIT, and colonoscopy every 10 years. However, this study found that screening with colonoscopy was more cost-effective than FIT. This was mainly owing to the sensitivity of FIT calculated in the two studies. The sensitivity of FIT for polyps and cancer was 62%, with the specificity was 93%. But the sensitivity of FIT for the detection of polyps in our model was much lower than that in Wong's. The results between two evaluations could be consistent if we used the same value after verification, suggesting the impact of instrument accuracy on the screening (32).

Recently, most economic evaluations on CRC screening placed emphases on fecal occult blood test and colonoscopy every 10 years in mainland China. But studies evaluating the impact of screening intervals of colonoscopy, starting age of screening and therapeutic effect of cancer treatment are lacking. Furthermore, some economic evaluations of CRC screening in China did not use ICER as an outcome indicator, resulting in incomparability with the international studies. This study analyzed the cost-effectiveness of CRC screening with FIT and colonoscopy following the standardized health economic evaluation procedures, addressing this important research gap. We also estimated the screening not only under the circumstance of the entire China, but also in a specific region. Moreover, we compared the results in different scenarios of various screening frequencies and starting ages, which is helpful for developing detailed strategies.

This study also has limitations. First, excluding the de novo and serrated lesion pathways may lead to overestimation of the cost-effectiveness of the screening. Second, the Markov model was unable to simulate the disease progress of distinct individuals, deviating from the real-world setting. Third, the study did not include direct non-medical costs and indirect costs because of data limitations. Therefore, it is difficult to evaluate screening programs from a societal perspective. Fourth, some parameters such as transition probabilities were derived from authoritative researches in other countries because local data was unavailable. But the deterministic analysis showed that the changes of these parameters would not influence the conclusions, and the results was robust in base-case analysis. At last, due to the lacking of relevant data, the impact of complex instrument and dedicated personnel required by the tests of colonoscopy was not considered which may cause the distinctions between the study and the real world. Despite the above limitations, this study provides evidence that is valuable for public health decision-making in China.

## Conclusions

It is cost-effective to implement CRC screening using FIT or electronic colonoscopy in mainland China, with FIT always saving costs. Additionally, colonoscopy is cost-effective compared with FIT, and a five-year interval is cost-effective compared with the 10 year interval, as the ICER was lower than the threshold of the GDP per capita of China in 2021 in all scenarios. Therefore, CRC screening is worth popularizing in China, and the economically developed regions such as Shenzhen could consider the strategy of electronic colonoscopy every 10 years, or even every 5 years.

## Data availability statement

The original contributions presented in the study are included in the article/Supplementary material, further inquiries can be directed to the corresponding author/s.

## Author contributions

Conceptualization: YR and MZ. Methodology: YR, MZ, and DZ. Validation: MZ, DZ, and WT. Formal analysis, investigation, software, and writing—original draft preparation: YR. Resources: YR and QX. Data curation: YR, MZ, and QX. Writing—review and editing: YR, MZ, and WT. Supervision: FG and WT. Funding acquisition: WT. All authors contributed to the article and approved the submitted version.

## Funding

This research was funded by General Program of National Natural Science Foundation of China (72174207), Key projects of National Natural Science Foundation of China (71734003), and Youth Project of National Natural Science Foundation of China (71603278).

## Conflict of interest

The authors declare that the research was conducted in the absence of any commercial or financial relationships that could be construed as a potential conflict of interest.

## Publisher's note

All claims expressed in this article are solely those of the authors and do not necessarily represent those of their affiliated organizations, or those of the publisher, the editors and the reviewers. Any product that may be evaluated in this article, or claim that may be made by its manufacturer, is not guaranteed or endorsed by the publisher.
