# Supplementary material for: Cost-effectiveness analysis of colonoscopy and fecal immunochemical testing for colorectal cancer screening in China
Source: Front Public Health. 2022 Aug 12;10:952378. doi: 10.3389/fpubh.2022.952378 (PMC9412186; doi:10.3389/fpubh.2022.952378)
Supplement: Supplementary file 1 [file Table_1.DOCX]

**Supplementary Table A.** Clinical parameters.

|  | **Parameters** | **Value** | **Lower value** | **Upper value** | **Distribution** | **Resource** |
| --- | --- | --- | --- | --- | --- | --- |
| Prevalence | Low-risk adenoma | 11% | 8.17% | 13.61% | Beta | Pan J, 2020  Sung J J, 2003 |
|  | High-risk adenoma | 7% | 5.41% | 9.01% | Beta |  |
|  | CRC | 0.216% | 0.20% | 0.23% | Beta | Yin J, 2019 |
| Prevalence of Shenzhen | Low-risk adenoma | 12.18% | 9.14% | 15.23% | Beta | Calculation |
|  | High-risk adenoma | 8.07% | 6.05% | 10.00% | Beta | Calculation |
|  | CRC | 0.15% | 0.11% | 0.19% | Beta | Calculation |
| The proportion of CRC | Stage I | 11.55% | 8.66% | 14.44% | Beta | Colorectal Cancer in 2019 |
|  | Stage II | 26.20% | 19.65% | 32.75% | Beta |  |
|  | Stage III | 32.67% | 24.50% | 40.84% | Beta |  |
|  | Stage IV | 29.44% | 22.08% | 36.80% | Beta |  |
| Visiting rate | Adenoma | 0% | - | - | Beta | Assumption |
|  | CRC I | 20% | 10% | 30% | Beta | Wong C K, 2015 |
|  | CRC II | 20% | 10% | 30% | Beta |  |
|  | CRC III | 65% | 45% | 85% | Beta |  |
|  | CRC IV | 100% | - | - | - | Assumption |
| Transition probability | Normal-low risk adenoma | 1.60% | 0.22% | 2% | Beta | Zhifang L,2017 |
|  | Low risk adenoma-high risk adenoma | 2% | 0.20% | 17.70% | Beta |  |
|  | High risk adenoma-CRC I | 4.40% | 0.50% | 6.30% | Beta |  |
|  | CRC I-II | 30% | 22.50% | 37.50% | Beta | Pickhardt P J, 2007 |
|  | CRC II- III | 45% | 33.75% | 56.25% | Beta | Hur C,2007 |
|  | CRC III- IV | 50% | 37.50% | 62.50% | Beta |  |
| Mortality | Adenoma | 0% | - | - | - | Assumption |
|  | CRC I | 0% | - | - | - | Tsoi K K, 2008 |
|  | CRC II | 1% | 0.75% | 1.25% | Beta |  |
|  | CRC III | 6% | 4.50% | 7.50% | Beta |  |
|  | CRC IV | 39% | 29.03% | 48.38% | Beta |  |
| Screening | Attendance rate-FIT | 60.00% | 45.00% | 75.00% | Beta | Assumption |
|  | Attendance rate-CSPY(FIT+) | 85.00% | 63.75% | 100.00% | Beta | Assumption |
|  | Attendance rate-CSPY | 61.25% | 45.94% | 76.56% | Beta | Calculation |
|  | Sensitivity-low risk adenoma-CSPY | 80% | 76.00% | 100.00% | Beta | Greuter M J, 2014 |
|  | Sensitivity-high risk adenoma-CSPY | 98% | 93.10% | 100.00% | Beta |  |
|  | Sensitivity-CRC-CSPY | 100% | 75.00% | 100.00% | Beta |  |
|  | Specificity-CSPY | 100% | - | - | Beta | Assumption |
|  | Sensitivity-low risk adenoma-FIT | 6.00% | 4.50% | 7.50% | Beta | Goede S L, 2013 |
|  | Sensitivity-high risk adenoma-FIT | 33.90% | 25.43% | 42.38% | Beta | Park D I, 2010 |
|  | Sensitivity-CRC-FIT | 84.60% | 63.75% | 100.00% | Beta |  |
|  | Specificity-FIT | 93.00% | 69.75% | 100.00% | Beta | de Wijkerslooth T R, 2012 |
|  | Resection rate | 100% | - | - | Beta | Assumption |
| Utility | Normal | 1 | - | - | - | Assumption |
|  | Low-risk adenoma | 0.871 | 0.751 | 0.991 | Beta | Wong C K, 2013 |
|  | High-risk adenoma | 0.832 | 0.712 | 0.952 | Beta |  |
|  | CRC I | 0.831 | 0.691 | 0.971 | Beta |  |
|  | CRC II | 0.858 | 0.738 | 0.978 | Beta |  |
|  | CRC III | 0.817 | 0.687 | 0.947 | Beta |  |
|  | CRC IV | 0.732 | 0.582 | 0.882 | Beta |  |
|  | Death | 0 | - | - | - | Assumption |
| Discount | Discount rate | 5% | 0% | 8% | Beta | Hongchao L, 2020 |

^1^ CRC I/ II/ III/ IV: colorectal cancer at stage I/ II/ III/ IV. CSPY-colonoscopy

Pan J, Cen L, Xu L, et al. Prevalence and risk factors for colorectal polyps in a Chinese population: a retrospective study[J]. Sci Rep, 2020,10(1):6974.

Sung J J, Chan F K, Leung W K, et al. Screening for colorectal cancer in Chinese: comparison of fecal occult blood test, flexible sigmoidoscopy, and colonoscopy[J]. Gastroenterology, 2003,124(3):608-614.

Yin J, Bai Z, Zhang J, et al. Burden of colorectal cancer in China, 1990-2017: Findings from the Global Burden of Disease Study 2017[J]. Chin J Cancer Res, 2019,31(3):489-498.

Colorectal Cancer in 2019[EB/OL]. [11-15]. https://www3.ha.org.hk/cancereg/pdf/factsheet/2019/colorectum_2019.pdf.

Wong C K, Lam C L, Wan Y F, et al. Cost-effectiveness simulation and analysis of colorectal cancer screening in Hong Kong Chinese population: comparison amongst colonoscopy, guaiac and immunologic fecal occult blood testing[J]. BMC Cancer, 2015,15:705.

Zhifang L, Huiyao H, Jufang S, et al. A systematic review of worldwide natural history models of colorectal cancer: classification, transition rate and a recommendation for developing Chinese population-specific model[J]. Chinese Journal of Epidemiology, 2017,38(02):253-260.

Pickhardt P J, Hassan C, Laghi A, et al. Cost-effectiveness of colorectal cancer screening with computed tomography colonography: the impact of not reporting diminutive lesions[J]. Cancer, 2007,109(11):2213-2221.

Hur C, Chung D C, Schoen R E, et al. The management of small polyps found by virtual colonoscopy: results of a decision analysis[J]. Clin Gastroenterol Hepatol, 2007,5(2):237-244.

Tsoi K K, Ng S S, Leung M C, et al. Cost-effectiveness analysis on screening for colorectal neoplasm and management of colorectal cancer in Asia[J]. Aliment Pharmacol Ther, 2008,28(3):353-363.

Greuter M J, Xu X M, Lew J B, et al. Modeling the Adenoma and Serrated pathway to Colorectal CAncer (ASCCA)[J]. Risk Anal, 2014,34(5):889-910.

Goede S L, van Roon A H, Reijerink J C, et al. Cost-effectiveness of one versus two sample faecal immunochemical testing for colorectal cancer screening[J]. Gut, 2013,62(5):727-734.

Park D I, Ryu S, Kim Y H, et al. Comparison of guaiac-based and quantitative immunochemical fecal occult blood testing in a population at average risk undergoing colorectal cancer screening[J]. Am J Gastroenterol, 2010,105(9):2017-2025.

de Wijkerslooth T R, Stoop E M, Bossuyt P M, et al. Immunochemical fecal occult blood testing is equally sensitive for proximal and distal advanced neoplasia[J]. Am J Gastroenterol, 2012,107(10):1570-1578.

Wong C K, Lam C L, Poon J T, et al. Clinical correlates of health preference and generic health-related quality of life in patients with colorectal neoplasms[J]. PLoS One, 2013,8(3):e58341.

Hongchao L, Guoen L, Shanlian H, et al. Guidelines for Pharmacoeconomic Evaluations 2020[M]. Beijing: China Market Press, 2020.
